# Opinion paper: Measuring livestock robustness and resilience: are we on the right track?

**DOI:** 10.1017/S1751731119003306

**Published:** 2020-01-09

**Authors:** P. Llonch, G. Hoffmann, R. Bodas, D. Mirbach, C. Verwer, M. J. Haskell

**Affiliations:** 1 Department of Animal and Food Science, Universitat Autònoma de Barcelona, Travessera dels Turons S/N, 08193 Cerdanyola del Vallès, Spain; 2 Department of Engineering for Livestock Management, Leibniz Institute for Agricultural Engineering and Bioeconomy, Max-Eyth-Allee 100, 14469 Potsdam, Germany; 3 Instituto de Ganadería de Montaña, Consejo Superior de Investigaciones Científicas–Universidad de León, Finca Zamadueñas, Ctra. Burgos km 119, 47071 Valladolid, Spain; 4 Deutsche Landwirtschafts-Gesellschaft e.V. – German Agricultural Society, Eschborner Landstrasse 122, 60489 Frankfurt/Main, Germany; 5 Louis Bolk Institute, Kosterijland 3-5, 3981 AJ Bunnik, The Netherlands; 6 Animal & Veterinary Sciences Research Group, Scotland’s Rural College (SRUC), King’s Building - West Mains Road, EH9 3JG Edinburgh, UK

## Introduction

Animal production is a cornerstone of employment and social structures in rural areas and, in some cases, it is highly dependent on the environment, which in a context of climate change makes it very vulnerable. For animal production systems to remain sustainable, this challenge must be managed to achieve economic stability and good welfare. The terms ‘robustness’ and ‘resilience’ are often used to describe a state of relative stability when animal production is confronted against global warming. However, the assessment of robustness and resilience in animal production systems has some gaps that need to be addressed prior to any definition of strategies to improve them. The methods to assess robustness and resilience have to be specific and provide a comprehensive and dynamic assessment. This opinion paper introduces these needs and suggests some potential solutions to tackle them, using dairy production as example as it is one of the animal production systems most exposed to forecasted environmental changes endangering feed scarcity, emergence of new diseases and increased heat stress.

## Robustness and resilience: all about adaptation capacity

Robustness and resilience have been sometimes used analogously. However, these two terms refer to different concepts. Resilience is the ability of an animal to adapt to stressors and its ability to return to the ‘normal’ state (Urruty *et al.*, 2016). Robustness, though, is the ability to express a high production potential combined with resilience to stressors in a wide variety of environmental conditions without compromising reproduction, health and welfare (Colditz and Hine, 2016). In short, robustness implies that the animal maintains functionality when a stressor is applied, which inevitably includes resilience. Both concepts, robustness and resilience, can apply not only to individual animals but also to higher organisational levels such as at the farm or system level. It refers to the adaptive capacity of a complex system to cope with disturbances and, at the same time, maintain its functions.

## Are robustness and resilience multidimensional or trait specific?

Robustness and resilience are often referred to as general characteristics of animals. Whereas this is applicable to robustness (Friggens *et al.*, 2017), resilience may be more specific to the type of stressor. For instance, it is possible that a cow that copes well with heat as a stressor may not manage so well with certain diseases. An example is the ‘slick’ hair gene that confers a degree of heat resistance for animals carrying that gene (Olson *et al.*, 2003), but it is not known (but not expected) that this gene will confer any resistance to infectious disease, as the characteristics that confer resilience to independent traits may not necessarily related to each other. Therefore, resilience is not considered to be a general trait in the sense that cows, or systems, are not more or less resilient but this has to be assessed for each particular trait. On the other hand, robustness may provide information about the individual animal or the system, which facilitates comparisons, based on multiple components including the resilience to each challenge. Considering this, robustness should be measured as a multidimensional attribute affecting either the whole animal or system, making comparisons between individuals or systems more plausible than resilience, as what is measured is the outcome rather than the relative response.

## Comprehensive assessment of robustness and resilience

Resilience can be assessed through the strategies triggered by the organism to cope with an environmental challenge (e.g. behaviour) and the consequences on the challenged organism (e.g. milk yield and quality). The coping strategies consist of the effort made by the animal to cope with the challenge, which can not only be behavioural, metabolic, physiological changes in the short term but can also result in morphological changes in the longer term. For instance, an animal challenged by heat can react rapidly by seeking shade, reducing metabolic energy expenditure and panting to cope with heat stress in the short term, whereas a longer term reaction could reduce the hair density on their skin. The consequences may vary among individuals not only in the magnitude of the response but also in the type of consequence. For instance, two cows exposed to heat stress will react differently, triggering different mechanisms to cope with the stressor. One cow might reduce feed intake to reduce heat production during rumen fermentation, whereas the other cow might reduce activity to decrease heat production from the skeletal muscles. Both strategies pursue the same goal, which is to reduce body temperature, but using different mechanisms. The consequences inform about the impact of the stressor to the organism irrespective of the adopted mechanisms to adapt to the challenge. An example of this would be the change in body temperature during heat load. For a comprehensive assessment of the adaptive capacity, both the coping strategies and the consequences should be addressed as they are interrelated. An incomplete assessment of the adaptive capacity based either on one mitigation trait (say activity or feeding behaviour) or a single consequence could lead to inaccurate conclusions as it risks of not reflecting the true coping cost for the animal. A refined determination of resilience should include a comprehensive phenotyping balancing the most active strategies to cope with a stressor against its consequences. Taking again the example of heat stress, the resilience towards heat can only be assessed by contrasting the increase in body temperature (i.e. the consequence) in relation to the strategies engaged in its mitigations, such as changes in feeding behaviour, reduction of metabolism and panting, to mention a few.

## Longitudinal assessment of robustness and resilience

Challenges to animals and systems vary in type and length. A challenge such as heat stress is typically of shorter duration than of poor housing that might last the lifetime of a cow.

In the definition of resilience discussed above, there is the concept that the animal, farm or system has the ability to ‘bounce back’ from stressors, which is especially relevant when they are short term. However, as robustness describes the capacity to withstand in harsh conditions, longer term scenarios are needed to be able to measure it. Irrespective of the duration of the challenge, we must observe the animal or system before, during and after the period of challenge to allow us to see the magnitude and the duration of the response. As an example, we could consider robustness in cow foot health. We can use a score to assess lameness in cows on two farms. Farm ‘A’ has very good conditions that might maintain good foot health (e.g. regular foot-bathing, good flooring, good cleanliness) and Farm ‘B’ has poor conditions (no foot-care, cracked concrete, not cleaned). On Farm ‘A’, the cows mostly have good feet and low lameness scores. Can we say that they are resilient in terms of lameness? Strictly speaking, we cannot, because we have not seen them challenged. On Farm ‘B’, we see cows that are lame and some that are not lame. We might conclude that non-lame cows are robust, because they have maintained functionality in a challenging situation, whereas lame cows are not. In order to assess the degree of robustness and resilience in individual animals or systems, we need to observe their adaptive response to changes in the environment, assessing them in good conditions and contrast this against the response to the challenge.

To assess the ability of an animal to respond and recover from challenge, some parameters are needed that can be measured easily, have been accurately validated, can be objectively assessed and ideally measured at any time point. Research studies have identified a number of these phenotypic traits on farms, slaughterhouses, etc. including behaviour, metabolism, physiology and anatomic characters. However, these phenotypic indicators have been typically used as ‘snapshot’ measures, whereas to assess robustness and resilience measures taken across the time course of a challenge are needed. The phenotype could be determined in experimental conditions by challenging an individual with some form of stressor and by subsequent longitudinal evaluation of various indicators over time. Along with research, data from commercial farms could be a precious source of information about this adaptive capacity to unplanned perturbations. Currently available technology based on precision livestock farming offers an enormous potential to gather information about the changes in the phenotype (both for adaptation strategies or consequences) due to commercially relevant stressors. These data may be extremely beneficial for producers and policy makers on the most adequate strategies to improve robustness and resilience (Berghof *et al.*, 2018), including breeding programmes and management actions.

## Actions needed for a reliable assessment

Integration of robustness into animal breeding programmes will improve the capacity of animal production systems to tackle the forecasted global change. Improving robustness inevitably implies increasing resilience towards environmental challenges. To improve robustness and resilience, reliable methods to monitor them should be considered, which will not be able to achieve unless a specific, comprehensive and longitudinal assessment is implemented. Specificity means that only the relevant traits associated with the adaptive response towards a thread should be considered. A comprehensive assessment means that the biological response towards a challenge has to balance the main adaptation strategies carried out by the animal (e.g. panting) and the consequences (e.g. body temperature) for the organism. A longitudinal assessment implies that animals have to be in constant monitoring that is able to inform about the evolution of the response against the threat. Current precision livestock farming tools offer the possibility to provide the amount of data necessary to comply with these demands. Progressive implementation of these tools in animal farming will inform farmers, breeding companies and policy makers about the hottest gaps that needs to be addressed to improve robustness and resilience in livestock systems.
